# How social preferences provide effort incentives in situations of financial support

**DOI:** 10.1371/journal.pone.0244972

**Published:** 2021-01-28

**Authors:** Christian Knoller, Stefan Neuß, Richard Peter

**Affiliations:** 1 Munich Risk and Insurance Center, Ludwig-Maximilians-Universität, München, Germany; 2 Department of Finance, University of Iowa, Iowa City, Iowa, United States of America; Universidad de Alicante, SPAIN

## Abstract

When people anticipate financial support, they may reduce preventive effort. We conjecture that the source of financial support can mitigate this moral hazard effect due to social preferences. We compare effort choices when another individual voluntarily provides financial support against effort choices under purely monetary incentives. When financial support is provided voluntarily by another individual, we expect recipients to exert more effort to avoid bad outcomes (level effect) and to reduce effort provision to a lesser degree as financial support becomes more generous (sensitivity effect). We conducted an incentivized laboratory experiment and find some evidence for the level effect and strong evidence for the sensitivity effect. This leads to significant gains in material efficiency with expected wealth being 5.5% higher and 37.3% less volatile.

## 1. Introduction

Financial support is commonly observed for a variety of risks such as natural disasters, economic dislocation, sickness and injury. There are private and public forms of financial support. Private forms of financial support are mostly evident after major world events, for example $2.4 billion in donations made to the victims of 9/11, $1.6 billion raised by US charities for disaster relief after the 2004 tsunami and $3.3 billion in cash raised after Hurricane Katrina [1]. Another example is financial support provided by a partner or family member, which is especially important in developing economies [2]. Public sources of financial support include governmental assistance to households after natural catastrophes and social security benefits to mitigate the financial impact of disease, disability, premature death and unemployment.

A common concern is that financial support may undermine risk-mitigation efforts. [3] model how moral hazard reduces welfare in the context of social security. According to [4], the provision of government relief is inefficient in situations where individuals can engage in self-protection because it reduces effort incentives. [5] reviews the history of natural disaster grant and loan programs in the US and argues that protecting citizens from the full consequences of their risky decisions leads to inefficiency and makes financial support self-perpetuating. According to [6], governmental relief programs crowd out the demand for private insurance. In terms of experimental research, [7] find moral hazard in a public goods context and [8] document increased risk-taking when others have to participate in losses. [9] provide an overview of experimental literature on moral hazard.

In this paper, we show how the voluntary provision of financial support by another individual preserves effort incentives. We attribute this effect to social preferences. If another individual shows kindness by promising financial support in case of a bad outcome, recipients reciprocate by maintaining high effort. As argued by [10], people have a propensity to be nice to those who treat them fairly but to punish those who hurt them. Reciprocity is a basic trait of human behavior [11,12]. Positive reciprocity can reinforce effort incentives in the presence of financial support.

The role of social preferences has mostly been discussed for giving behavior and incentive contracting. The economic analysis of giving behavior is cast within the realm of altruism and social preference theories [13–15] such as pure altruism [16], inequality aversion [15,17], impure altruism [18] and conditional altruism [19]. The perspective of recipients of financial support has hardly been analyzed so far. Social preferences also play a key role in contracting. Reciprocity enlarges the set of enforceable contracts resulting in large efficiency gains [20]. Several papers focus on the principal’s perspective and analyze how specific forms of social preferences change the effectiveness of alternative incentive schemes. When agents are fair-minded, principals prefer less complete contracts such as bonus contracts over more complete contracts [21–23]. Others have studied distributional concerns and reciprocity [24], inequality aversion [25], envy [26], inequity aversion [27] and co-worker altruism [28] in optimal contracting. [29] provides a survey.

We investigate effort provision in situations where people voluntarily provide financial support to others who face the risk of an unfavorable outcome. Consistent with the contracting literature, we conjecture that anticipation of financial support undermines effort incentives but less so whenever one’s choice affects another individual. This is due to social preferences of the recipient of financial support towards its provider. We decompose this moral-hazard mitigating effect into a level effect, predicting higher effort under social preferences, and a sensitivity effect, predicting effort to be less sensitive to changes in financial support under social preferences. So we distinguish between level and slope of the moral-hazard mitigating effect.

To test our predictions, we conducted a controlled laboratory experiment with two treatments. Participants were exposed to a binary risk and received either low or high income. We varied the outcome in the low-income state between participants. The better the low-income state, the lower the incentive to invest in costly effort ex ante. In the social treatment we implemented financial incentives with the help of financial support, which was provided voluntarily by another participant. Financial incentives in the private treatment were identical in strength to the social treatment but given by design. We argue that social preferences can only be present in the social treatment. The participants display moral hazard only if incentives are provided by design. The better off individuals are in the low-income state, the less they invest in protective effort. If the improvement in the low-income state results from the voluntary provision of financial support by another individual, moral hazard cannot be detected and the relationship between financial incentives and effort is significantly less negative. This leads to significant gains in material efficiency with expected wealth being 5.5% higher and 37.3% less volatile.

We conclude that the implementation of financial support is an important design variable, which has the potential to reduce the inefficiency arising from moral hazard in the giver-receiver relationship. Our findings provide an economic efficiency rationale for sponsorship programs, where donors and recipients are matched in a bilateral relationship. Recipients do not receive support from an abstract fund or organization, but know about the voluntary nature of their donor’s contribution and may even know their benefactor personally. Such matching may also help to recruit donors and increase their generosity because it can dissipate concerns of shirking. More generally, our results have implications for policy-makers who aspire to provide financial support without undermining effort provision. Whenever it is possible to make transparent that actual people provided money for financial support, this personal link should reduce free-riding and improve economic efficiency. Based on our results, moral hazard is more likely when recipients associate financial support with abstract entities and not with other individuals.

## 2. Theoretical predictions

We use a simple model to analyze how financial support affects effort provision and how this effect interacts with social preferences. We assume risk-neutral preferences over consumption. due to the small stakes in the experiment [30]. Consider an individual who is exposed to a binary risk. Her consumption is *x*_*H*_ in the high-income state with probability *p*(*e*), or *x*_*L*_<*x*_*H*_ in the low-income state with probability (1−*p*(*e*)). The probabilities depend on the individual’s effort level $e\in [0,\bar{e}]$ such that *p*′(*e*)>0 and *p*′′(*e*)≤0. Effort is costly with cost function *c*(*e*) such that *c*′(*e*)>0 and *c*′′(*e*)≥0. The individual’s objective function is then given by

$$U(e)=p(e)x_{H}+(1-p(e))x_{L}-c(e),$$

and optimal effort is characterized by the associated first-order condition,

$$U_{e}=p^{\prime }(e)[x_{H}-x_{L}]-c^{\prime }(e)=0$$

for an interior solution. The second-order condition is satisfied. Optimal effort trades off the marginal benefit of higher expected consumption due to a lower likelihood of being in the low-income state against the marginal cost of lower consumption due to the effort investment.

Suppose now that the individual anticipates financial support of *λ*>0 in the low-income state. We assume *x*_*L*_+*λ*≤*x*_*H*_ so that financial support does not make the individual better off than if the loss had not happened. The first-order condition for optimal effort *e*^0^ changes to

$$U_{e}=p^{\prime }(e^{0})[x_{H}-x_{L}-\lambda ]-c^{\prime }(e^{0})=0.$$

Optimal effort is now lower than before because financial support diminishes the marginal benefit of effort. Furthermore *U*_*eλ*_ = −*p*′(*e*^0^)<0, so more generous financial support reduces effort.

If the individual receives financial support of *λ*>0 *from another individual*, this may activate social preferences towards the provider of financial support. A possible rationale is positive reciprocity [10] because the recipient feels treated kindly by the giver who offers to help out in case of a bad outcome. To reciprocate, the recipient may choose a high effort level to avoid needing financial support altogether. Another rationale is pure altruism towards the giver [18] because the giver’s likelihood of having to follow through with her promised support depends on the individual’s effort choice. We subsume these considerations as social preferences and let parameter *α* ∈ (0,1) measures their intensity. *α* is bounded by one to exclude cases where the recipient weighs the giver’s welfare higher than her own, which seems unrealistic. This assumption could easily be relaxed. Parameter *α* allows us to incorporate the effect of the recipient’s effort choice on the giver’s welfare into the recipient’s effort trade-off. If the giver’s income is *w*, the recipient’s objective function in the presence of social preferences is

$$V(e)=p(e)x_{H}+(1-p(e))(x_{L}+\lambda )-c(e)+\alpha [p(e)w+(1-p(e))(w-\lambda )],$$

and optimal effort e* solves the associated first-order condition

$$V_{e}=p^{\prime }(e^{*})[x_{H}-x_{L}-\lambda ]-c^{\prime }(e^{*})+\alpha \lambda p^{\prime }(e^{*})=0$$

for an interior solution. The second-order condition holds. The recipient now has an additional incentive to invest in effort arising from her social preferences towards the provider of financial support. We thus formulate our first hypothesis, which is about the effect of social preferences on effort provision at the extensive margin.

### 2.1. Hypothesis 1a (H1a)

Effort provision in the presence of social preferences is higher than in the absence of social preferences (*level effect*).

Our model also predicts that effort provision is positively associated with the intensity of social preferences (i.e., with *α*) because *V*_*eα*_ = *λp*′(e*)>0. If more generous financial support induces stronger social preferences in the recipient, the difference in effort provision between a situation with and without social preferences depends on the level of financial support. This motivates our next hypothesis about the intensive margin.

### 2.2. Hypothesis 1b (H1b)

The difference in effort provision with and without social preferences is larger at high levels of financial support than at low levels of financial support.

Besides the two level effects we also investigate how sensitive effort provision is to changes in financial support. Recall that *e*^0^ and e* denote optimal effort in the absence and in the presence of social preferences. The implicit function theorem yields

$$\frac{de^{*}}{d\lambda }=-\frac{V_{e\lambda }}{V_{ee}}=-\frac{\left(\alpha -1)p^{\prime }(e^{*}\right)}{p^{\prime \prime }(e^{*})[x_{H}-x_{L}-\lambda ]-c^{\prime \prime }(e^{*})+\alpha \lambda p^{\prime \prime }(e^{*})}<0,$$

and likewise for *e*^0^. More generous financial support lowers effort provision consistent with the notion of moral hazard. To evaluate how this effect is mitigated by social preferences, we compare the slope of optimal effort with respect to financial support for a positive *α* (i.e., de*/d*λ*) and the slope of optimal effort with respect to financial support when *α* is zero (i.e., d*e*^0^/d*λ*). In S1 Appendix, we show that *p*′′′(*e*)≤0 and *c*′′′(*e*)≥0 are jointly sufficient for our next hypothesis, which is about the extensive margin effect of social preferences on effort sensitivity.

### 2.3. Hypothesis 2a (H2a)

Effort provision is less sensitive to changes in financial support in the presence of social preferences than in the absence of social preferences (*sensitivity effect*).

We also study the strength of the sensitivity effect for different intensities of social preferences, the intensive margin. The two conditions *p*′′′(*e*)≤0 and *c*′′′(*e*)≥0 are also jointly sufficient for $\frac{\partial }{\partial \alpha }\frac{de}{d\lambda }\geq 0$. In this case, stronger social preferences flatten the relationship between effort provision and financial support. If more generous financial support induces stronger social preferences in the recipient, the sensitivity effect is more pronounced at high levels of financial support than at low ones. We formulate this as our last hypothesis.

### 2.4. Hypothesis 2b (H2b)

The difference between the sensitivity of effort provision to changes in financial support with and without social preferences is larger at high levels of financial support than at low levels of financial support.

**Fig 1:**
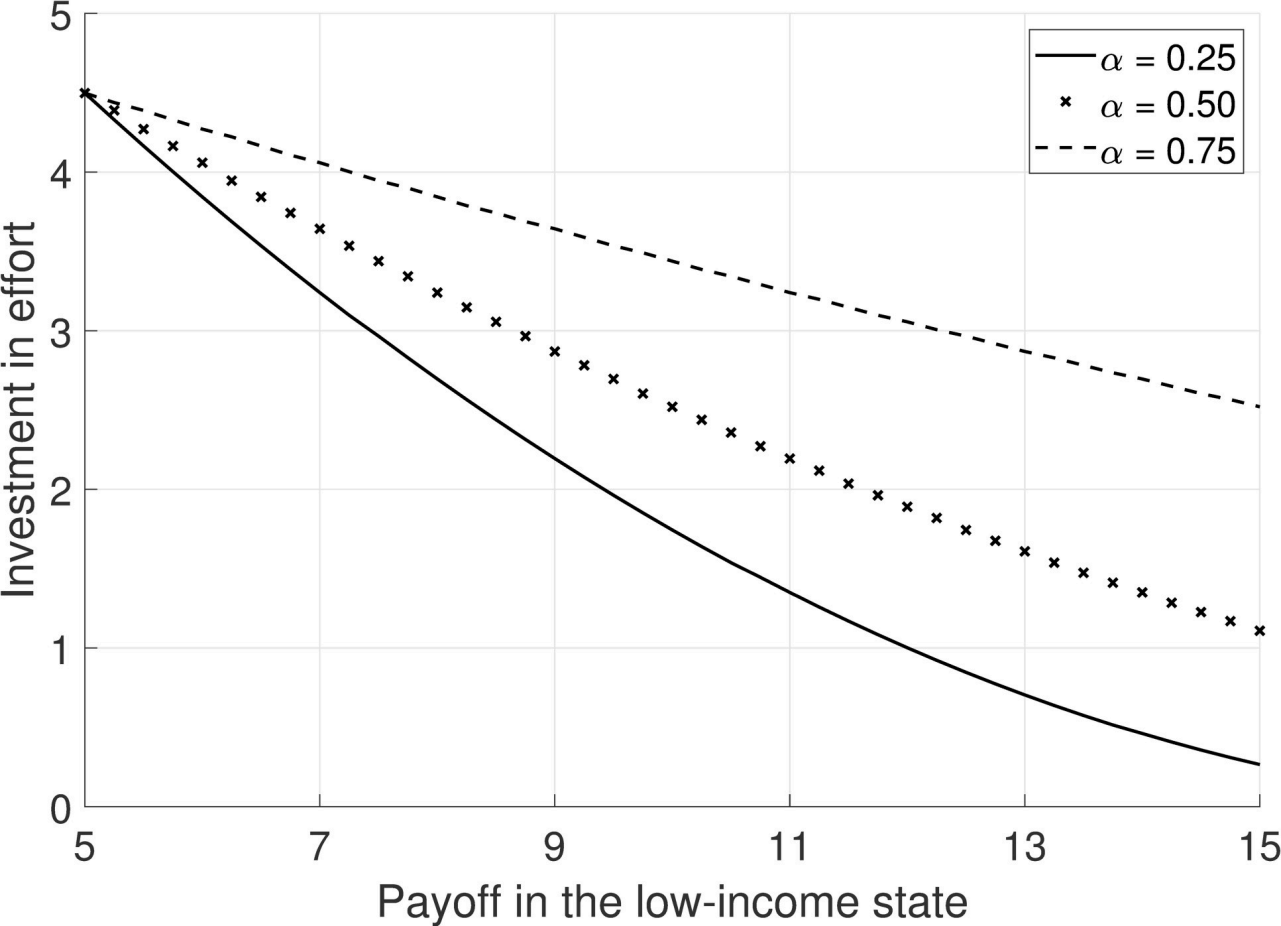
Effort provision as a function of financial support for different intensities of social preferences α.

To provide more intuition for our hypothesis, we visualize the relationship between the payoff in the low-income state and the effort investment for different levels of social preferences in Fig 1. We use the parameters from the experiment with *x*_*H*_ = 15, *x*_*L*_ = 5 and *λ* ranging from 0 to 10. We replace the discrete probability and cost functions from the experiment with their continuous counterparts to obtain smooth curves. Increasing the intensity of social preferences (i.e., higher *α*) leads to higher investment in effort (H1a) and a flatter curve (H2a). If high levels of financial support induce stronger social preferences (i.e., going from *α* = 0.25 to *α* = 0.75 instead of from *α* = 0.25 to *α* = 0.50), the effort investment increases by more (H1b) and the curve becomes even flatter (H2b) compared to low levels of financial support.

[31] find higher effort provision by agents when principals donate. Unlike in our study the principal’s donation goes to an external third party and does not affect the agents directly. [20] show how reciprocity helps enforce contracts and leads to higher gains from trade, especially when both parties to the contract can reciprocate. If we interpret the donor as the principal and the recipient as the agent, this theory predicts the level effect in our paper (hypothesis H1a). Existing literature is silent about the sensitivity effect (hypothesis H2a) and the intensity of both effects in social preferences at different levels of financial support (hypotheses H1b and H2b).

## 3. Experimental design

We conducted an incentivized laboratory experiment to test our hypotheses. We randomly allocated participants to either the role of “subject X” or “subject Y”. Subject Y individuals were endowed with EUR 5 and participated in a risky lottery, which returned EUR 10 with probability *p*(*e*) or nothing with probability (1−*p*(*e*)). If the lottery returned zero, subject Y individuals received an amount *λ* ∈ [EUR 0; EUR 10], which varied between participants. This amount can be interpreted as financial support or insurance to protect against the bad outcome, even though the experiment was neutrally framed (i.e., the terms “financial support” or “insurance” were not used). The payoff in the high-income state is then EUR 15 and the payoff in the low-income state is EUR 5 + *λ*. Higher amounts of *λ* represent more generous financial support. In the real world recipients do not know *λ* for sure at the time of their effort choice. We did not include uncertainty over the size of *λ* in the experiment to reduce confounds. After subject Y individuals observed this amount, they determined the probability distribution over states of the world by investing in effort, which was deducted from their endowment. The relationship between the cost of effort and the resulting probabilities is presented in Table 1. The probability of the high-income state increases linearly in effort, consistent with *p*′(*e*)>0 and *p*′′(*e*)≤0, while the cost function is increasing with increasing marginal cost, consistent with *c*′(*e*)>0 and *c*′′(*e*)≥0.

**Table 1:** Cost of effort, marginal cost of effort and associated probabilities.

Cost of effort (in EUR)	0.00	0.20	0.60	1.20	1.80	2.40	3.20	4.00	5.00
Marginal cost of effort (in EUR)	0.00	0.20	0.40	0.60	0.60	0.60	0.80	0.80	1.00
Probability of high-income state	10	20	30	40	50	60	70	80	90
(in percentage points)									
Probability of low-income state	90	80	70	60	50	40	30	20	10
(in percentage points)									

We implemented two treatments, which differed exclusively in the provision of incentives, see S2 Appendix for a visualization. In the **social treatment**, *λ* was a voluntary transfer made by another individual (subject X). Subjects were randomly matched into pairs consisting of one subject X and one subject Y. All subject X individuals were endowed with EUR 20, which was also known by their matched subject Y. Subject X individuals were then told that their subject Y was endowed with EUR 5 and faced a lottery of winning EUR 10 with probability *p*(*e*) or nothing with probability (1−*p*(*e*)). Subject X knew that their matched subject Y can make a costly effort choice after learning about the transfer to raise the probability of the high-income state. Subject X individuals also knew subject Y’s effort technology and cost function. Each subject X had the option to specify a transfer *λ* ∈ [EUR 0; EUR 10] to her subject Y, which was paid conditional on the occurrence of the low-income state. This transfer decision was made before subject Y’s effort choice and before the resolution of uncertainty. The transfer was then deducted from subject X’s endowment only if the low-income state actually occurred. No transfer could be made for the high-income state. It was made clear to each subject X that her matched subject Y first observes the transfer and afterwards chooses an effort level. We provide an overview of the transfers made by subject X individuals in S3 Appendix.

In the **private treatment**, *λ* was implicitly included in the experimental design. All participants in this treatment were assigned the role of subject Y. The subjects were told that, if the low-income state occurs, they will receive the sum of *x*_*L*_ and *λ*. To compare the behavior of subject Y individuals between the two treatments, we assigned this role to the same number of participants in each treatment. Subject Y individuals do not differ in observables across treatments (difference in gender: p = 0.848; difference in age: p = 0.843). We conducted the social treatment first and then used the observed *λ* amounts to calibrate the private treatment. Therefore, the distribution of *λ* amounts was the same in both treatments. This ensures that financial incentives for effort provision are identical in both treatments. The only difference is that, in the social treatment, financial incentives are implemented with the help of financial support which was provided *voluntarily by another individual*; in the private treatment, financial incentives arise by design and do not involve another individual.

Our decision to tell subjects only the aggregate of *x*_*L*_ and *λ* in the private treatment but to inform them about the different roles of *x*_*L*_ and *λ* in the social treatment is deliberate. [32] identify social preferences towards the experimenter as a possible confound in the laboratory. They find that giving to the experimenter was intermediate between giving to a charity and giving to another subject [33,34]. Communicating only the sum of *x*_*L*_ and *λ* to subject Y individuals in the private treatment mutes experimenter demand effects. This minimizes any perception on behalf of subject Y individuals that they are being treated kindly or being bailed out by the experimenter, which allows us to focus solely on financial incentives in the private treatment. We argue that, in real life, when people anticipate financial support by an abstract entity (i.e., the government, an insurance company, a social welfare program), they are unlikely to exhibit social preferences in the form of positive reciprocity or pure altruism towards the support-providing entity but instead make decisions based on private costs and benefits.

Using a between-subjects design participants were randomly allocated to either the social treatment or the private treatment. They were fully informed about the experimental design of the treatment they were allocated to, so subject Y individuals knew what determined the financial incentives they faced and specifically whether their payoff in the low-income state involved another individual’s decision to provide financial support to them or not. Subject Y individuals were not informed about the existence of the other treatment. The experiment was approved by the Munich Experimental Laboratory for Economic and Social Science (MELESSA, www.melessa.uni-muenchen.de) and conducted in November 2014 and January 2015 with the experimental software *z-tree* [35]. The participants were recruited through the Online Recruitment System for Economic Experiments (ORSEE) by [36]. The participants’ decisions in the experiment and chance determined how much they earned, and the laboratory paid the money.

We conducted the social treatment first. After arriving at the laboratory, the participants were randomly allocated to a seat in front of a computer and matched into pairs. In each pair one participant was subject X and the other one was subject Y. The matched subjects were not introduced to each other and did not know with whom they had been paired. Each transfer that was specified by a subject X to her matched subject Y in the social treatment was randomly assigned to one of the subjects participating in the private treatment. In both treatments all instructions were shown on the computer and all participants could take as much time as they needed to read the instructions and ask as many questions as they wanted. After reading the instructions, the participants had to answer some questions individually to verify that they comprehended the procedure of the experiment, their options, and how payoffs were determined. The questionnaire ensured that all subjects understood the experiment. They could only start the experiment once all questions were answered correctly. The participants consented to the use of the experimental data in electronic form.

162 individuals participated in the experiment, 108 in the social treatment and 54 in the private treatment. All participants in the private treatment were assigned the role of subject Y. In the social treatment 54 participants were randomly assigned the role of subject Y and 54 participants were randomly assigned the role of subject X. 60 percent of the participants were female and 40 percent were male. The majority of participants (91 percent) were students at one of Munich’s universities. The average age was 25 years with a minimum age of 18 and a maximum age of 69.

## 4. Experimental results

### 4.1. Statistical analysis

We conduct various parametric tests to analyze our hypotheses and use non-parametric tests as robustness checks. S4 Appendix presents a scatterplot of the experimental data. We first compare individuals who did not receive any transfer in the social treatment and their counterparts in the private treatment (N = 30). Based on our model, we do not expect differences in behavior between treatments for those individuals. The p-value of a t-test is high (p = 0.7501) and so are the p-values of a Wilcoxon rank-sum test (p = 0.9831) and a Kolmogorov-Smirnov test (p = 0.999). As expected, we cannot reject the null hypothesis of identical behavior across treatments when no transfer was received.

In a next step we compare subject Y individuals whose payoff in the low-income state exceeds EUR 5.00 so that their behavior affects subject X’s welfare in the social treatment. This leaves 78 individuals, 39 in the social treatment and 39 in the private treatment, and we report the mean, standard deviation and median of their effort investment in the first row of Table 2 (*Overall*). We also report results if we split the sample in subjects whose payoff in the low-income state is below average (*λ*<3) and in subjects whose payoff in the low-income state is at least the average (*λ*≥3). Other splits produce similar directional results but the sample size becomes small. Overall, mean and median effort levels are higher in the social treatment than the private treatment but the difference is not significant so there is no support for H1a. While the difference between effort investments also fails to be significant among subjects with a below-average payoff in the low-income state, the social treatment provides significantly higher effort incentives than the private treatment for subjects with an above-average payoff in the low-income state. We construe this as evidence in support of H1b. The amount of financial support can be interpreted as a measure of the giver’s generosity. If more generous giving activates stronger social preferences in the recipients of financial support, this explains why we only observe significant differences in effort incentives for above-average transfers. These results are confirmed when using a Wilcoxon rank sum test. There is no significant difference in effort incentives for the overall sample (p = 0.193) or for subjects with a below-average payoff in the low-income state (p = 0.574), whereas effort incentives are significantly higher in the social treatment than the private treatment for subjects with an above-average payoff in the low-income state (p = 0.055). The difference in the last comparison is only significant at the 10% level and just fails to be significant at the 5% level.

**Table 2:** Effort provision by subject Y individuals in both treatments.

	Social treatment			Private treatment				
	Mean	Std. dev.	Median	Mean	Std. dev.	Median	t-test	p-value
Overall	3.026	1.470	3.2	2.497	1.786	2.4	1.426	0.158
*λ*<3	3.462	1.152	3.6	3.477	1.637	4.0	-0.039	0.969
*λ*≥3	2.793	1.575	2.4	1.950	1.561	1.8	2.011	0.049

We now analyze how effort provision reacts to changes in financial incentives. We calculate Pearson’s correlation coefficient between the payoff in the low-income state and the investment in effort. We report our results in Table 3. In the social treatment, the correlation coefficients are all positive and insignificant for the overall sample and the split samples. As soon as another individual is involved in the implementation of financial incentives, the null of no moral hazard can no longer be rejected. This lack of a significant negative effect is consistent with our expectation that social preferences preserve incentives for effort provision even though financial effort incentives become weaker. In the private treatment instead, the correlation coefficients are negative and significant at the 5% level for the overall sample and for individuals with an above-average payoff in the low-income state. This confirms that, from a purely monetary standpoint, individuals would indeed find it optimal to provide less effort. The lack of significance for individuals with a below-average payoff in the low-income state is probably due to the limited variation in payoffs for *λ*<3. If we use Spearman’s rank correlation or Kendall’s tau as measures of association between payoffs in the low-income state and effort investments, the same picture emerges. All measures are positive and insignificant in the social treatment for the overall and the split samples, but they are negative and significant in the private treatment for the overall sample and for individuals with an above-average payoff in the low-income state.

**Table 3:** Pearson’s correlation between payoffs in the low-income state and effort provision.

	Social treatment				Private treatment			
	Correlation coefficient	p-value	95% confidence interval		Correlation coefficient	p-value	95% confidence interval	
Overall	0.008	0.961	-0.309	0.322	-0.563	<0.001	-0.733	-0.274
*λ*<3	0.228	0.263	-0.192	0.553	0.292	0.148	-0.130	0.596
*λ*≥3	0.271	0.163	-0.133	0.572	-0.459	0.014	-0.693	-0.070

In Fig 1 each value of *α* corresponds to a curve with a particular slope in the payoff-effort investment plane. Based on the cost and probability functions in the experiment, the lowest possible slope is -0.534 in the absence of social preferences for *α* = 0, which is close to Pearson’s correlation coefficient of -0.563 reported in Table 3 in the private treatment. The highest possible slope is zero for *α* = 1, which indicates that recipients care as much about the providers of financial support as they care about themselves. Pearson’s correlation coefficient of 0.008 with a p-value of 0.961 in the social treatment is virtually indistinguishable from zero.

To assess whether the differences between treatments are significant, we reiterate the correlation coefficients and report Fisher’s r-to-z transformation in Table 4. Overall, the correlation between the payoff in the low-income state and the investment in effort is significantly higher in the social treatment than the private treatment at the 5% level and even at the 1% level, which confirms H2a. When financial support is provided voluntarily by another individual, effort provision is significantly less sensitive to changes in financial incentives than if individuals make effort decisions purely based on monetary considerations. The split sample shows that this is driven by individuals who receive high transfers in the social treatment. For individuals with a below-average payoff in the low-income state, the difference in effort sensitivities is insignificant. As explained above, for those individuals there is no significant relationship between the payoff in the low-income state and effort provision in either treatment. However, when individuals face an above-average payoff in the low-income state, the treatment effect is significant at the 5% level and even at the 1% level. We construe this as evidence of H2b.

**Table 4:** Fisher’s r-to-z transformation to test for significant differences between correlation coefficients across treatments.

	Correlation coefficient		Fisher’s r-to-z transformation	
	Social	Private	z-score	p-value
Overall	0.008	-0.563	2.737	0.006
*λ*<3	0.228	0.292	-0.243	0.810
*λ*≥3	0.271	-0.459	2.625	0.009

An alternative approach to evaluate the significance of the difference between two correlation coefficients is the method by [37]. It allows us to calculate confidence intervals for the difference between two independent correlations from their respective confidence intervals. For the overall sample, the 95% confidence interval for the difference between the two correlations is [0.142, 0.928] and excludes zero. For individuals with a below-average payoff in the low-income state, the 95% confidence interval for the difference between correlations is [-0.583, 0.469] and contains zero whereas it is [0.169, 1.111] and thus excluding zero for individuals with an above-average payoff in the low-income state. These non-parametric results are in line with the results based on Fisher’s r-to-z transformation. The differences between correlations are significant even at the 1% level for the overall sample and for individuals with an above-average payoff in the low-income state when using the method by [37], which corroborates the support of H2a and H2b. In S5 Appendix, we also report the results of a Tobit regression that controls for observables. The effects are by and large consistent with our previous findings.

### 4.2. Discussion

Our results identify a difference in the reaction of subject Y individuals to effort incentives between treatments. As payoffs in the low-income state increase, individuals provide significantly less effort when incentives are given by design (moral hazard). As soon as financial incentives involve voluntary provision of financial support by another individual, moral hazard can no longer be detected. Differences across treatments are significant. The split sample reveals that our results are driven by the behavior of individuals who experience generous financial support relative to the rest of the sample. We argue that such differences originate from the intensity of social preferences, which is higher in giver-receiver relationships with a generous giver.

In [8] subjects received financial support because other subjects were forced to participate in losses. They find evidence of moral hazard which shows the crucial role of the voluntary nature of financial support in our set-up. The different findings suggest that our results are mostly driven by positive reciprocity instead of pure altruism. In [8] and in our set-up individuals know that their behavior affects others and that increased risk-taking or reduced precaution raise the likelihood of others experiencing losses. If individuals were pure altruists, both set-ups should lead to the same conclusion. In our paper, however, the givers provide financial support *voluntarily*, which can activate positive reciprocity in the recipients. When participation in losses is involuntary as in [8], the provision of financial support is not a deliberate decision of its provider, and there is no ground for reciprocity. An alternative explanation for our results is warm glow utility on behalf of recipients [18]. As with pure altruism, this explanation is less convincing than positive reciprocity because the level and sensitivity effect are more pronounced at high levels of financial support than low ones.

Another possible effect is that the provision of financial support may affect the level of risk aversion of the recipients. [38] find a tendency towards less risk aversion while [39,40] document lower loss aversion when people decide for others. So if anything, this channel appears to suggest less effort, not more, although the theoretical link between risk aversion and effort is complicated [41,42]. [43] argue that such effects require knowledge of the other participant’s risk preferences, which is not the case in our set-up. Furthermore, the stakes in the experiment are small so that risk aversion is unlikely to play an important role [30]. The interaction between social and risk preferences in settings of financial support is a fascinating topic for further research.

We now compare the outcomes of both treatments in terms of material efficiency. We use pre-support expected wealth (EW) of subject Y individuals to compare the induced effort distributions across treatments. This criterion arises naturally in the social treatment by determining aggregate wealth of each giver-receiver pair:

$$\left(1-p(e))(\left[w-\lambda ]+[x_{L}+\lambda -c(e)\right])+p(e)(w+[x_{H}-c(e)]\right)$$

$$=w+\underset{\mathrm{subject}\;Y's\;\mathrm{pre}‐\mathrm{support}\;\mathrm{expected}\;\mathrm{wealth}}{\underset{︸}{x_{L}∙(1-p(e))+x_{H}∙p(e)-c(e).}}$$

EW is maximal for the effort level that does not presuppose financial support. Given the cost and probability function in the experiment, EW is strictly increasing in effort and peaks at the highest possible effort level. In Table 5, we report the mean and standard deviation of EW for individuals with a positive transfer in the social treatment and their counterparts in the private treatment. EW is 5.5% higher (8.513 versus 8.067) and 37.3% less volatile (0.727 versus 1.160) in the social treatment than in the private treatment. The difference in means is significant at the 5% level and the difference in standard deviations is significant at the 1% level. We conclude that effort provision via social preferences leads to gains in material efficiency because the welfare of giver-receiver pairs is significantly higher and significantly less volatile compared to situations where receivers make effort choices purely based on monetary considerations.

**Table 5:** Comparison of material efficiency across treatments.

	Mean	Std. dev.	Test	t-test	F-test
Social treatment	8.513	0.727	Test statistic	2.035	0.393
Private treatment	8.067	1.160	p-value	0.046	0.003

## 5. Conclusion

We conducted a laboratory experiment to analyze how effort provision is affected by financial support. We compare a treatment where financial incentives are given by design and a treatment where they arise via the voluntary provision of financial support by another individual. A simple model predicts that social preferences mitigate moral hazard via two channels, a level effect and a sensitivity effect, both of which are stronger at higher levels of financial support. Our experimental results support these hypotheses. There is some evidence for the level effect and strong evidence for the sensitivity effect. The voluntary provision of financial support induces individuals to reciprocate in terms of their effort choice. This social effect is strong enough to make moral hazard undetectable. It is driven by recipients who experience generous support.

Our results have practical implications. They provide an economic efficiency rationale for matching donors to recipients. Moral hazard may be a concern for individuals who issue a guarantee or stand surety in favor of another individual. Beneficiaries might interpret the guarantee as free insurance coverage and exert less effort. According to our results moral hazard will not be an issue in such situations. This effect appears to be particularly relevant in developing economies where formal insurance and safety net mechanisms are often absent and household members use informal forms of self-insurance to insulate each other from income shocks. Our findings suggest that such arrangements are not plagued by moral hazard compared to more institutionalized settings, where individuals anticipate to receive financial support by design.

More generally, charitable giving, social insurance and governmental support face a design trade-off. Charity is based on the idea that philanthropic individuals transfer money to others who suffered a major loss or have fallen on hard times. Stronger institutionalization may lower transaction costs and increase the efficiency of operations. However, it may intensify the moral hazard problem. Recipients will see individual donors behind the money they are receiving less clearly. It is easier for them to free-ride the system than to free-ride on individual donors. This caveat may prevent potential donors from making contributions altogether. On the other hand, voluntary and less institutionalized financial support will frequently be less efficient in terms of transaction costs and operational efficiency. However, it may help mitigate moral hazard problems. This trade-off suggests that informal and voluntary financial support can be a solution in situations where otherwise financial support would not be viable due to moral hazard.

Our results share some of the common limitations of laboratory experiments in economics. People may react differently in the lab than in real life. According to [44], social behavior in the lab and in the field correlate strongly. Most of our subjects were college students (91 percent). For social preferences, the behavior of students is not significantly different from the general population [45]. If anything, students are slightly more selfish [12,46] but this difference may depend on their familiarity with lab experiments [47]. Finally, the participants may have behaved differently had the stakes been higher. According to [48] and [49], an increase in stakes has small effects on the intensity of social behavior.

We suggest some avenues for further research. We muted experimenter demand effects by telling individuals the aggregate of *x*_*L*_ and *λ* in the private treatment. It would be interesting to measure the strength of experimenter demand effects in situations of financial support directly. Second, we elicited the amount of financial support upfront and informed the recipients accordingly. In the real world, the recipients’ expectations about financial support affect effort choices because financial support is often uncertain. A treatment where donors have the possibility to renege on their promise could help elicit how this affects the recipients’ belief formation and effort provision. Finally, we matched individuals into pairs in the social treatment but they were not personally introduced to each other. There is evidence that pro-social behavior becomes stronger the lower the social distance between individuals [50–52]. How the degree of anonymity affects effort choices in situations of financial support is an interesting topic for future research.

## Supporting information

S1 Appendix Sufficient conditions for hypotheses H2a and H2b. (DOCX)

S2 Appendix Visualization of treatments (translated from German). (DOCX)

S3 Appendix Overview of transfers made by subject X individuals. (DOCX)

S4 Appendix Scatterplot of experimental data. (DOCX)

S5 Appendix Tobit model. (DOCX)
