# Supplementary material for: How social preferences provide effort incentives in situations of financial support
Source: PLoS One. 2021 Jan 28;16(1):e0244972. doi: 10.1371/journal.pone.0244972 (PMC7842880; doi:10.1371/journal.pone.0244972)
Supplement: S1 Appendix — (DOCX) [file pone.0244972.s001.docx]

**S1 Appendix: Sufficient Conditions for Hypotheses H2a and H2b**

To derive H2a, notice that

$$\frac{\text{d}e^{0}}{\text{d}\lambda}=\frac{p^{'}\left( e^{0} \right)}{p^{''}\left( e^{0} \right)\left[ x_{H}-x_{L}-\lambda\right]-c^{''}\left( e^{0} \right)}\leq\frac{\left( 1-\alpha\right)p^{'}\left( e^{*} \right)}{p^{''}\left( e^{*} \right)\left[ x_{H}-x_{L}-\lambda\right]-c^{''}\left( e^{*} \right)+\alpha\lambda p^{''}\left( e^{*} \right)}=\frac{\text{d}e^{*}}{\text{d}\lambda}$$

if and only if

$$\left[ x_{H}-x_{L}-\lambda\right]\cdot\left[ p^{'}\left( e^{0} \right)p^{''}\left( e^{*} \right)-\left( 1-\alpha\right)p^{'}\left( e^{*} \right)p^{''}\left( e^{0} \right) \right]$$

$$+\left[ \left( 1-\alpha\right)p^{'}\left( e^{*} \right)c^{''}\left( e^{0} \right)-p^{'}\left( e^{0} \right)c^{''}\left( e^{*} \right) \right]+\alpha\lambda p^{'}\left( e^{0} \right)p^{''}\left( e^{*} \right)\leq0.$$

The first square bracket is non-negative from the definition of$\lambda$; the second square bracket is non-positive if$p^{'''}(e)\leq0$; the third square bracket is non-positive if$c^{'''}(e)\geq0$; the fourth term is non-positive in any case. So $p^{'''}(e)\leq0$ and $c^{'''}(e)\geq0$ are jointly sufficient for effort to be more sensitive to changes in financial support in the absence of social preferences than in their presence. Neither of the two conditions is individually necessary for social preferences to reduce effort sensitivity with respect to changes in financial support. The assumption $p^{'''}(e)\leq0$ may seem at odds with $p^{'}(e)>0$ and$p^{''}(e)\leq0$ but it is satisfied for the linear effort technology in the experiment. An alternative sufficient condition that permits $p^{'''}\left( e \right)>0$ is that $-{p^{''}\left( e \right)}/{p^{'}\left( e \right)}$ is non-decreasing in effort, which is equivalent to weak log-concavity of$p^{'}\left( e \right)$, see [53].

With a similar argument $p^{'''}(e)\leq0$ and $c^{'''}(e)\geq0$ are jointly sufficient for $\frac{\partial}{\partial\alpha}\frac{\text{d}e}{\text{d}\lambda}\geq0$, which is hypothesis H2b. Our hypotheses continue to hold if preferences over consumption are assumed risk-averse instead of risk-neutral, see [54].
