# Supplementary material for: How social preferences provide effort incentives in situations of financial support
Source: PLoS One. 2021 Jan 28;16(1):e0244972. doi: 10.1371/journal.pone.0244972 (PMC7842880; doi:10.1371/journal.pone.0244972)
Supplement: S2 Appendix — (DOCX) [file pone.0244972.s002.docx]

**S2 Appendix: Visualization of Treatments (Translated from German)**

Social Treatment:

**Participant X has specified a transfer of EUR 4.00 in the case that state B occurs.**

Your payment situation can be depicted as follows:

**State A**

EUR 15 minus your
chosen investment

**State B**

EUR 9 minus your
chosen investment

Participant Y

Please choose an investment now. Each investment results in specific costs for your account and determines the probabilities of State A and State B. The relationship between the investment and the respective probabilities of State A and State B can once again be found in the table below.

**Reminder:**

If State A occurs, you receive EUR 15 minus your chosen investment. If State B occurs, you receive EUR 9 minus your chosen investment

**Please choose an investment now. Note that values of Euros and Cents in this program are separated by dots, not commas, as usual in German.**

**Your choice is relevant for your payment!**

Investment Euro

Private Treatment:

Your payment situation can be depicted as follows:

**State A**

EUR 15 minus your
chosen investment

**State B**

EUR 9 minus your
chosen investment

Please choose an investment now. Each investment results in specific costs for your account and determines the probabilities of State A and State B. The relationship between the investment and the respective probabilities of State A and State B can once again be found in the table below.

**Reminder:**

If State A occurs, you receive EUR 15 minus your chosen investment. If State B occurs, you receive EUR 9 minus your chosen investment

**Please choose an investment now. Note that values of Euros and Cents in this program are separated by dots, not commas, as usual in German.**

**Your choice is relevant for your payment!**

Investment Euro
