# Supplementary material for: How social preferences provide effort incentives in situations of financial support
Source: PLoS One. 2021 Jan 28;16(1):e0244972. doi: 10.1371/journal.pone.0244972 (PMC7842880; doi:10.1371/journal.pone.0244972)
Supplement: S3 Appendix — (DOCX) [file pone.0244972.s003.docx]

**S3 Appendix: Overview of Transfers made by Subject X Individuals**

Fig 2 provides a frequency graph of transfers made by subject X individuals. About a quarter of subject X individuals transferred nothing to their subject Y. Most subject X individuals transferred a positive amount not exceeding EUR 5.00. Only a few subject X individuals selected more than EUR 5.00. The mean transfer is EUR 2.99 and is virtually identical to the median of EUR 3.00. The standard deviation is EUR 2.69. The observed giving behavior is similar to other dictator-game experiments [55]. The fact that subject X individuals may not have to follow through with their intended transfer in case the high-income state occurs did not seem to affect the overall distribution of transfers compared to the literature. The exact motives underlying subject X individuals’ giving behavior are irrelevant for our study because we focus on the recipients’ effort provision.


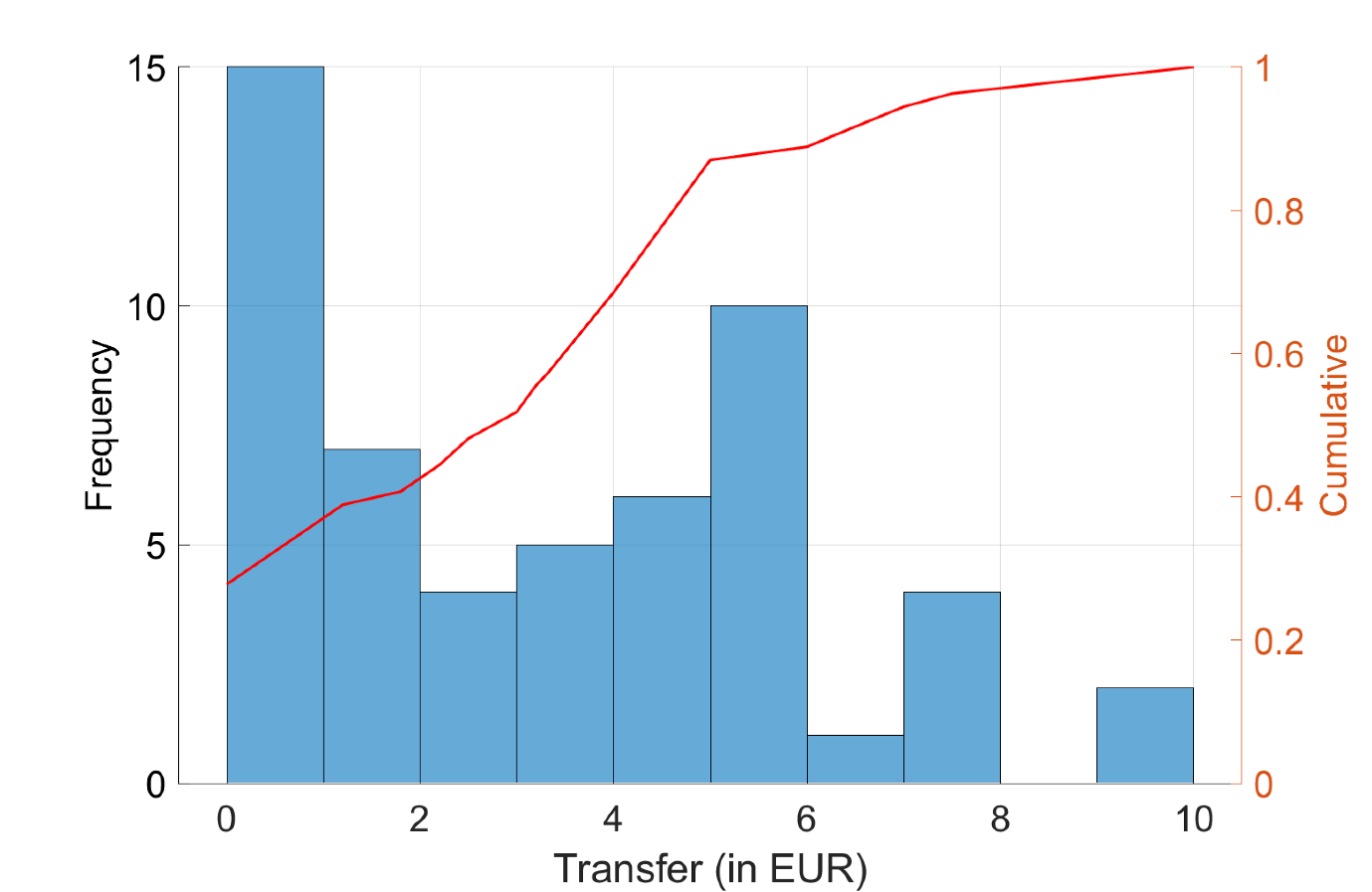


Fig 2. Frequency graph of transfers made by subject X individuals.
