# Supplementary material for: How social preferences provide effort incentives in situations of financial support
Source: PLoS One. 2021 Jan 28;16(1):e0244972. doi: 10.1371/journal.pone.0244972 (PMC7842880; doi:10.1371/journal.pone.0244972)
Supplement: S4 Appendix — (DOCX) [file pone.0244972.s004.docx]

**S4 Table: Scatterplot of Experimental Data**

In Fig 3 we plot subject Y individuals’ investment in effort against the payoff in the low-income state. The size of the bubbles represents the number of observations for each combination of payoff and effort. As explained in the text, the variation in the payoffs in the low-income state was identical in both treatments because we conducted the social treatment first and then used the observed transfers to calibrate the private treatment


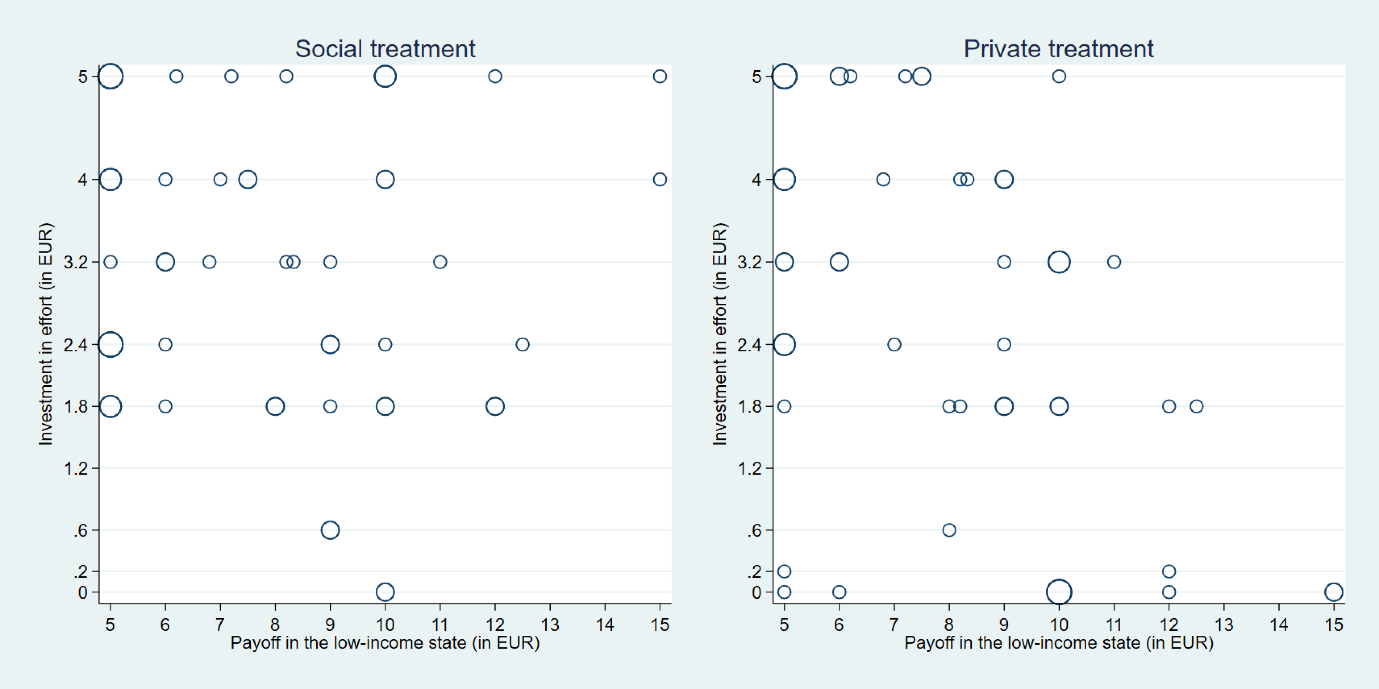


Fig 3. Scatterplots of effort investments against the payoff in the low-income state for both treatments.
