# Supplementary material for: How social preferences provide effort incentives in situations of financial support
Source: PLoS One. 2021 Jan 28;16(1):e0244972. doi: 10.1371/journal.pone.0244972 (PMC7842880; doi:10.1371/journal.pone.0244972)
Supplement: S5 Appendix — (DOCX) [file pone.0244972.s005.docx]

**S5 Appendix: Tobit Model**

We estimate a Tobit model of effort provision in Table 6. The Tobit model is suitable because the investment in effort is bounded from above and below. We include a treatment dummy variable (Private), the lottery payoff in the low-income state (Payoff_low_), and an interaction term between the two (Private x Payoff_low_) as explanatory variables to identify financial incentives. We estimate the model with and without controls for the subjects’ age and gender. The coefficient for the private treatment is positive and significant, the coefficient for the payoff in the low-income state is positive but insignificant, and the coefficient for the interaction term is negative and significant. When evaluated at the payoffs used in the experiment, average effort provision does not differ significantly across treatments but participants reacted to variations in financial incentives more strongly in the private treatment than in the social treatment.

| Tobit Model | | | |
| --- | --- | --- | --- |
| VARIABLES |  | Investment in effort | |
| Private |  | 6.003*** (2.116) | 6.117*** (2.135) |
| Payoff_low_ |  | 0.030 (0.148) | 0.039 (0.148) |
| Private x Payoff_low_ |  | -0.742*** (0.228) | -0.751*** (0.230) |
| Female |  |  | 0.420 (0.486) |
| Age |  |  | 0.028 (0.025) |
| Constant |  | 2.932  (1.388) | 1.886  (1.572) |
|  |  |  |  |
| Observations |  | 78 | 78 |
| Standard errors in parentheses *** p<0.01, ** p<0.05, * p<0.1 | | | |

Table 6: Tobit model of effort provision by subject Y individuals.

**Supplementary References**

[53] Courbage C, Loubergé H, Peter R. Optimal Prevention for Multiple Risks. Journal of Risk and Insurance. 2017; 84(3): 899-922.

[54] Knoller C, Neuß S, Peter R. How Social Preferences Mitigate Moral Hazard in Situations of Financial Support. Working Paper, University of Iowa, Department of Finance. 2018; [http://dx.doi.org/10.2139/ssrn.3257434.](http://dx.doi.org/10.2139/ssrn.3257434.%20)

[55] Engel, C. Dictator Games: A Meta Study. Experimental Economics. 2011; 14(4): 583-610.
